# CDKN2A inhibits formation of homotypic cell-in-cell structures

**DOI:** 10.1038/s41389-018-0056-4

**Published:** 2018-06-05

**Authors:** Jianqing Liang, Jie Fan, Manna Wang, Zubiao Niu, Zhengrong Zhang, Long Yuan, Yanhong Tai, Zhaolie Chen, Santai Song, Xiaoning Wang, Xiaoqing Liu, Hongyan Huang, Qiang Sun

**Affiliations:** 1 Department of Oncology, Beijing Shijitan Hospital of Capital Medical University, 10 TIEYI Road, 100038 Beijing, P. R. China; 2 Institute of Biotechnology, 20 Dongda Street, 100071 Beijing, P.R. China; 3 School of Biological Science and Engineering, South China University of Technology, 510000 Guangzhou, P.R. China; 4 The 307 Hospital, 8 Dongda Street, 100071 Beijing, P. R. China

## Abstract

Cell-in-cell (CIC) structures, characterized by enclosure of one or more cells within another cell, were extensively documented in human cancers. Although elevated CIC formation was found in cancers with CDKN2A inactivation, a causal link between them remains to be established. We reported here that inhibiting CDKN2A expression effectively promoted homotypic CIC formation, whereas ectopic overexpression of p16INK4a or p14ARF, two proteins encoded by CDKN2A gene, significantly suppressed CIC formation in MCF7 cells. The regulation of CIC formation by CDKN2A was tightly correlated with subcellular redistribution of E-cadherin, F-actin rearrangement and reduced phosphorylation of myosin light chain 2 (p-MLC2), consistent with which, CDKN2A expression imparted cells winner/outer identity in competition assay. Moreover, CIC formation negatively correlates with p16INK4a expression in human breast cancers. Thus, our work identifies CDKN2A as the first tumor suppressor whose inactivation promotes homotypic CIC formation in human cancer cells.

## Introduction

A panel of human cancer tissues displayed unique cell-in-cell (CIC) structures^1^, which were often associated with worse prognosis^2,3^. Homotypic CIC structures formation involves the invasion of one viable cell into another, which generally leads to the death of internalized cells in a non-apoptotic way that was termed Entosis^4^. Researches on entosis revealed that actomyosin contraction within the internalizing cells driven the formation of CIC structures^4,5^, which also requires intercellular adhesion mediated by adherens junction (AJ)^6^. Although loss expression of AJ components, such as E-cadherin, P-cadherin and α-catenin, found a common way for cancer cells to escape entotic cell death mediated by homotypic CIC formation^6,7^, little is known about the genetic controls that initiate the formation of CIC structures in human cancers.

Cyclin-dependent kinase inhibitor 2A (CDKN2A), located on 9p21 locus, is a well-established tumor suppressor that was frequently inactivated in multiple human tumors, including melanomas, glioblastomas, pancreatic cancers, bladder cancers and the like^8–10^. The CDKN2A gene encodes two important cell cycle regulators: p16INK4a and p14ARF proteins, the former plays an executional role in cell cycle and senescence mainly through the regulation of the CDK 4/6 and cyclin D complexes, whereas the later regulates cell cycle by blocking MDM2-induced degradation of p53 to enhance p53-dependent transactivation^11^. Recently, Matsumoto et al.^12^ reported that mesothelioma cells with 9p21 homozygous deletion exhibited significantly more CIC structures than those with intact 9p21 loci. However, it is unknown whether 9p21 deletion and CIC formation are two parallel events or they are causatively linked. Interestingly, MCF7 cells, the entosis-competent cells that were routinely used for CIC research, are also deleted in 9p21 loci leading to loss of CDKN2A. We therefore hypothesized that genes affected by 9p21 deletion, such as CKDN2A, might be responsible for increased CIC formation.

## Results

### Reduced CDKN2A expression promotes CIC formation

**Fig. 1:**
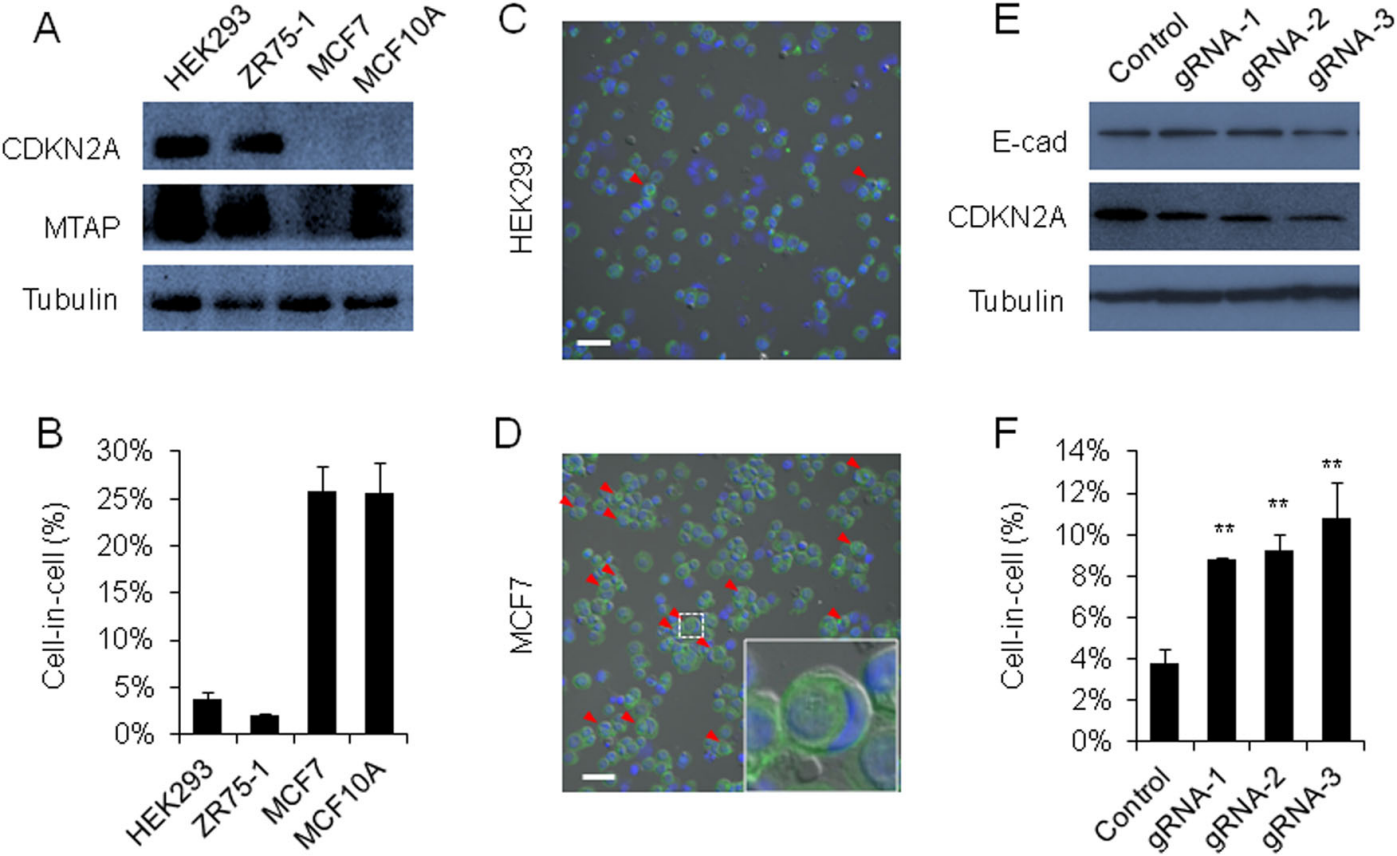
Reduced CDKN2A expression promotes CIC formation. **a** Expression of endogenous CDKN2A and MTAP in different cell lines by western blot. Tubulin was used as loading control. **b** CIC frequency in different cell lines. Cells were cultured in suspension for 6 h or 12 h (HEK293) before analysis. Data are mean ± SD of three or more fields with >600 cells analyzed for each cell line. **c**, **d** Representative cytospin images for HEK293 cells (**c**) and MCF7 cells **d**. Cells were stained with phalloidin in green to show F-actin and DAPI in blue for nuclei. Red arrows indicate internalized cells of CIC structure. Scale bar: 100 μm. **e** Expression of E-cadherin (E-cad) and CDKN2A in CDKN2A knock-down HEK293 cells by western blot. Three gRNAs were used. Tubulin is loading control. **f** Quantification of CIC structures in CDKN2A knock-down HEK293 cells. Cells were cultured in suspension for 12 h before analysis. Data are mean ± SD of three or more fields with >600 cells analyzed for each cell line. **p < 0.01

To test the role of 9p21 deletion on CIC formation, we examined expression of CDKN2A and MTAP, two neighboring genes that are frequently affected by 9p21 deletion in most human cancers^8,13^, in HEK293, ZR75-1, MCF7 and MCF10A cells. As shown in Fig. 1a–d, although CDKN2A expression could be readily detected in two low-CIC cell lines (HEK293 and ZR75-1), it is undetectable in human breast cancer cell MCF7 and non-transformed mammary epithelial cell MCF10A, two cell lines that could form high frequency of CIC structures, suggesting a negative role of CDKN2A in CIC formation. Consistently, knocking down CDKN2A expression, by three different gRNAs via CRISPR/Cas9-mediated gene editing (Fig. 1e), significantly promoted CICs formation in HEK293 cells (Fig. 1f). As for MTAP, although MCF7 cells displayed marginal expression, MCF10A cells expressed considerable amount of MTAP protein. Therefore, it is unlikely that MTAP directly regulates CIC formation in these two cells.

### Ectopic expression of CDKN2A inhibits CIC formation

**Fig. 2:**
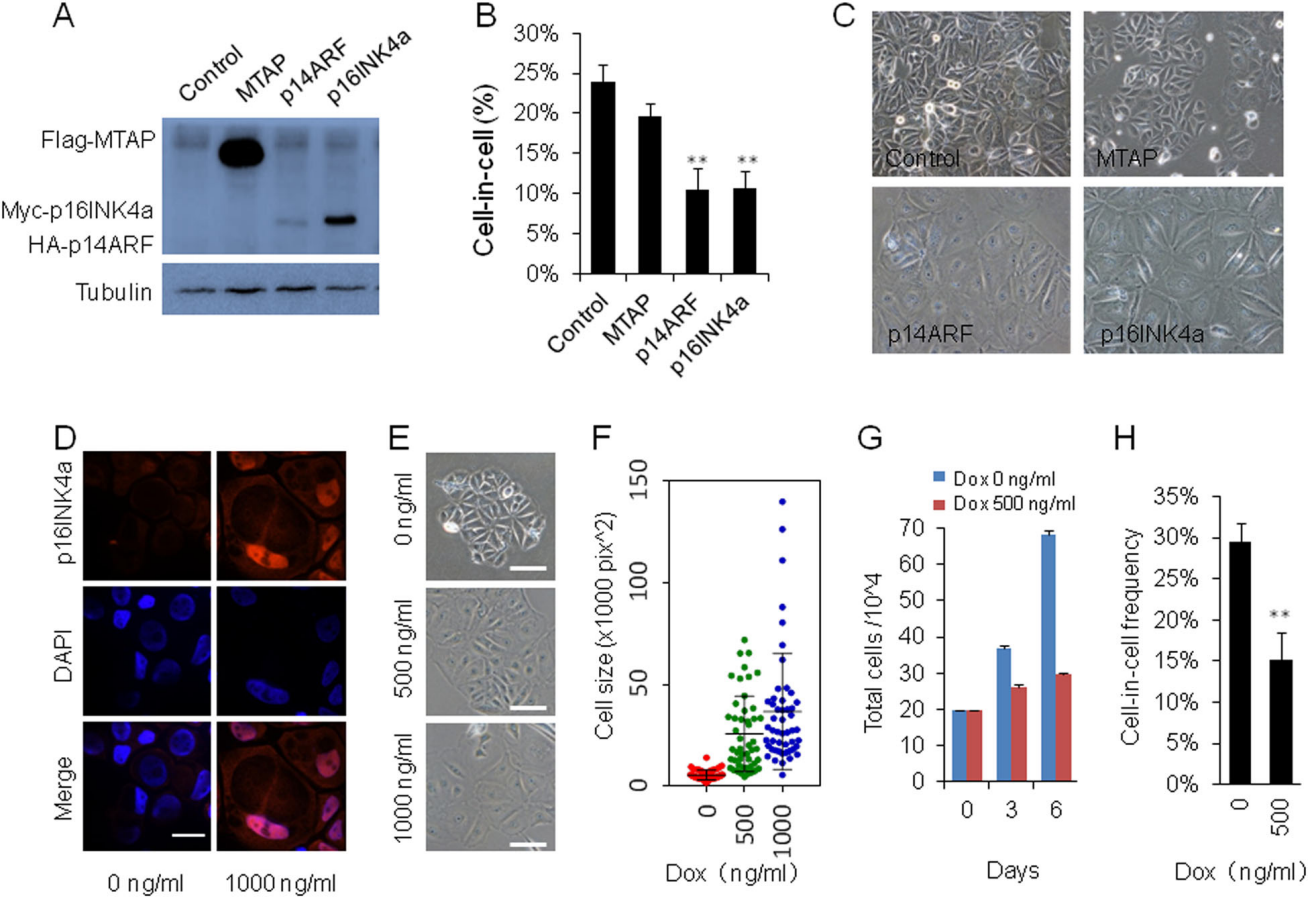
Ectopic expression of CDKN2A inhibits CIC formation. **a** Expression of p14ARF, p16INK4a and MTAP in MCF7 cells infected with respective retroviruses. Western blot was performed with mixed antibodies against different tags. **b** CIC formation in MCF7 cells overexpressing MTAP, p14ARF and p16INK4a. Data are mean ± SD of three or more fields with >600 cells analyzed for each cell line. **p < 0.01. **c** Representative images for MCF7 cells overexpressing MTAP, p14ARF and p16INK4a. **d** Inducible expression of p16INK4a in MCF7 cells by immunostaining. Scale bar: 30 μm. **e** Morphology changes of MCF7 cells upon Dox-induced expression of p16INK4a. Scale bar: 30 μm. **f** Increased cell size upon Dox-induced expression of p16INK4a; (*n* = 50 for each). **g** Cell growth was inhibited in cells expressing p16INK4a. **h** CIC formation was inhibited in MCF7 cells upon Dox-induced expression of p16INK4a. Data are mean ± SD of three or more fields with >600 cells analyzed for each cell line. **p < 0.01

To further confirm the role of CDKN2A in CIC formation, we expressed exogenous Myc-tagged p16INK4a, HA-tagged p14ARF and FLAG-tagged MTAP as well, respectively, by retroviral vector in MCF7 cell line. As evidenced by western blot (Fig. 2a), these genes were successfully expressed. Importantly, cells expressing p14ARF or p16INK4a displayed compromised CIC formation (Fig. 2b), meanwhile, MTAP seemly had little impact despite robust expression (Fig. 2b), supporting the notion that 9p21 deletion primes cells to undergo CIC formation via loss expression of CDKN2A (p16INK4a/p14ARF) genes. In agreement, overexpressing p16INK4a or p14ARF in MCF10A cells, another CIC-proficient cell line, also significantly inhibited CIC formation (data not shown).

As persistent CDKN2A expression was known to induce senescence^14^. To rule out that the effect of CIC inhibition was secondary to senescence, we employed the Tet-on advanced Doxycycline (Dox)-inducible expression system to control CDKN2A gene expression. MCF7 cells were sequentially infected with the regulatory plasmid (pLVX-Lenti-X Tet-On Advanced) and response plasmid (pLVX-Tight-p16INK4a and pLVX-Tight-p14ARF) to make stable cell lines. As shown in Fig. 2d, p16INK4a could be readily detected in cell nuclei and cytoplasm in the presence of DOX for 48 h when senescence had not occurred yet. Expression of p16INK4a resulted in dramatic morphology changes as evidenced by bigger cell size (Fig. 2c–f), and growth inhibition (Fig. 2g). Moreover, CIC formation was also inhibited as did in MCF7 cells with constitutive CDKN2A expression (Fig. 2b, h). Inducible expression of p14ARF gave phenotypes resembling those of p16INK4a (data not shown). Together, the data above support a negative role of CDKN2A in regulating CIC formation.

### Redistribution of E-cadherin and F-actin in CDKN2A-expressing cells

**Fig. 3:**
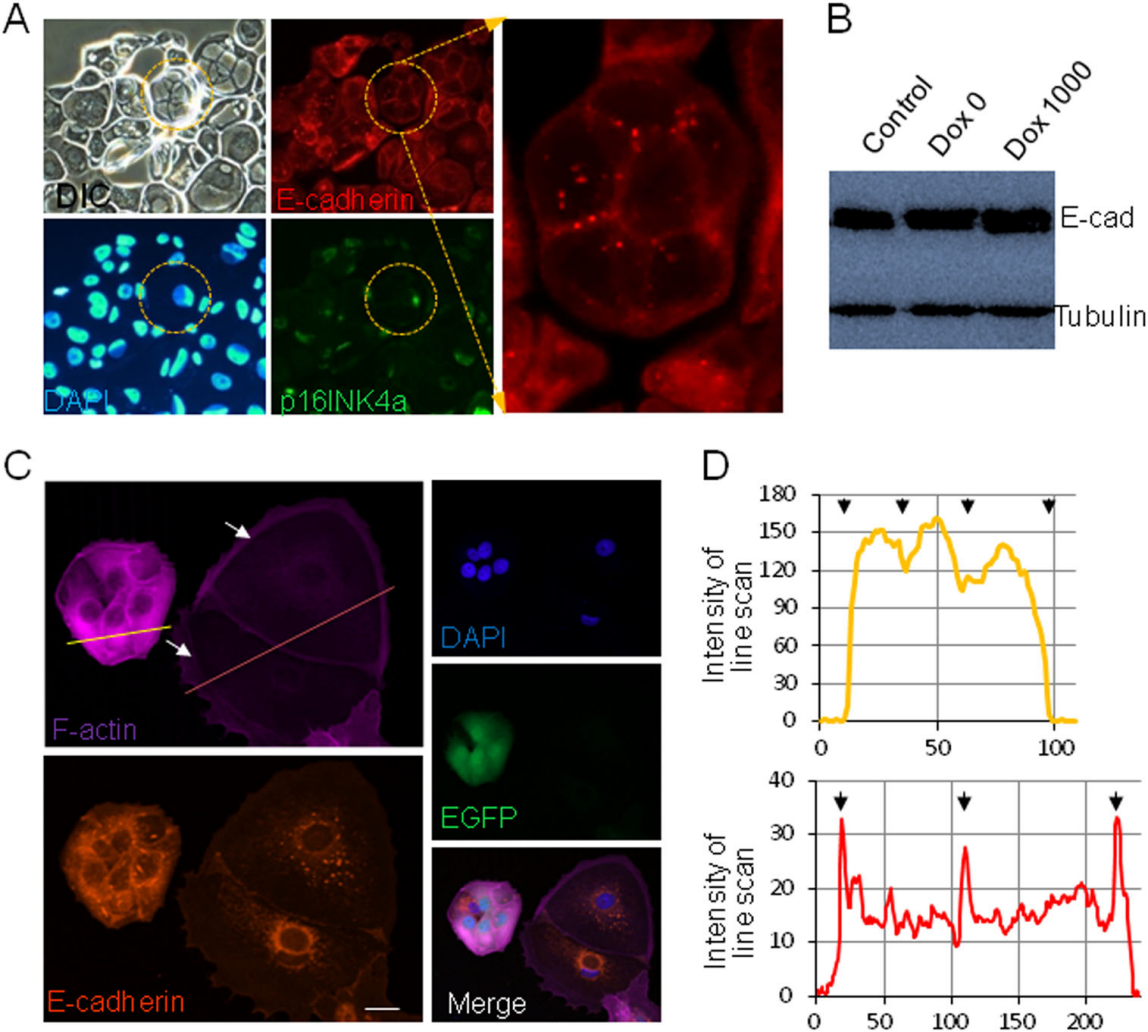
Redistribution of E-cadherin and F-actin in CDKN2A-expressing cells. **a** Subcellular localization of E-cadherin in suspended MCF7 cells expressing p16INK4a. **b** E-cadherin expression upon Dox-induced expression of p16INK4a in MCF7 cells. **c** Subcellular localization of F-actin and E-cadherin in control (EGFP-positive cells) and p16INK4a-expressing MCF7 cells (arrows indicated cells). Scale bar: 10 μm. **d** Intensity of F-actin across line-scanned cells in **c**, arrows indicated boundaries of cells

**Fig. 4:**
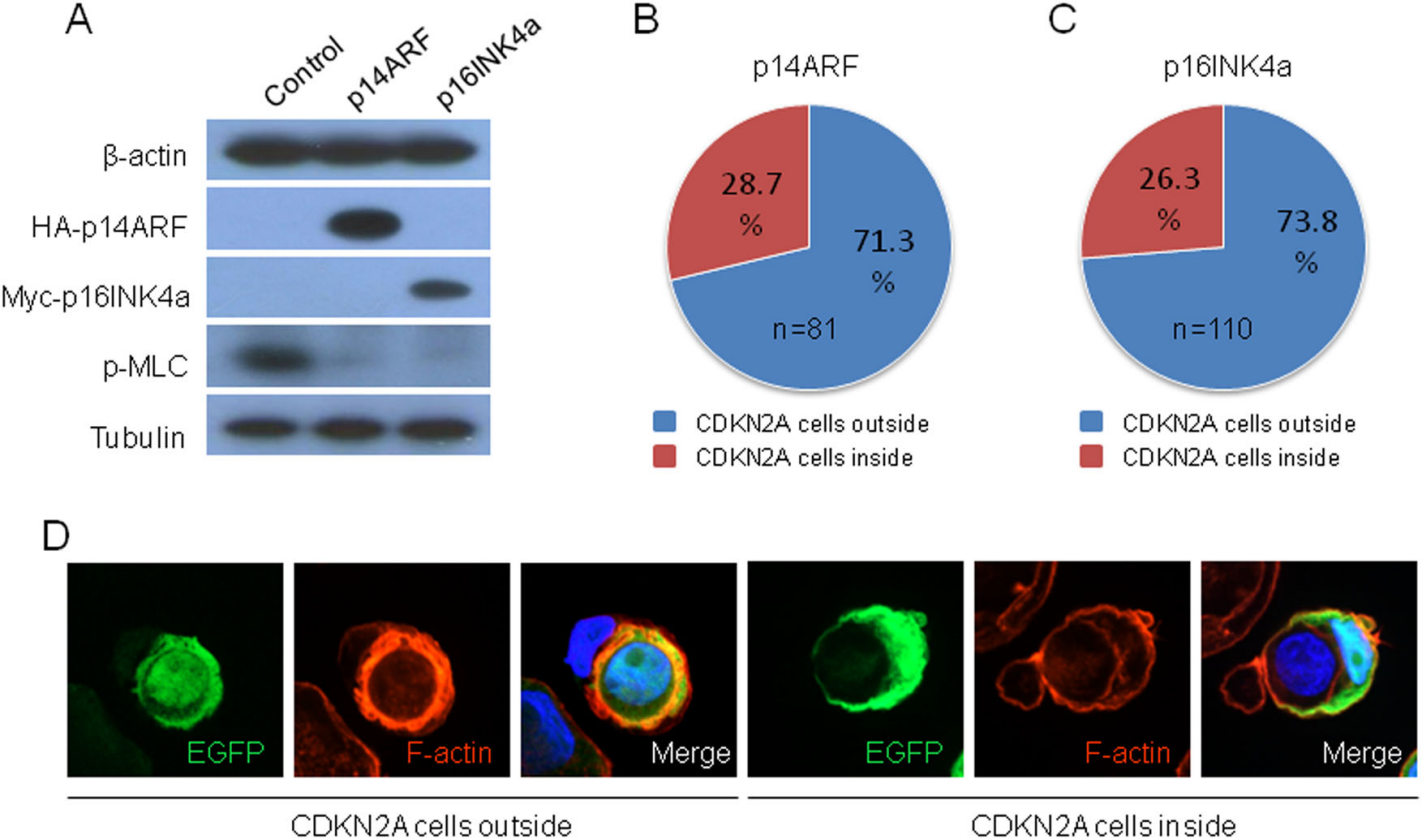
CDKN2A confers cells winner identity. **a** CDKN2A inhibits p-MLC expression as detected by western blot. Tubulin was used as loading control. **b**, **c** Position analysis in CIC structures formed between control and cells expressing p14ARF (B, *n* = 81) or p16INK4a (**c**, *n* = 110). **d** Representative images of CIC structures formed between control (EGFP-positive) and CDKN2A-expressing cells

Our previous work showed that cell–cell adhesion mediated by epithelial cadherin (E-cadherin) is essential to form CICs, we therefore examined the expression and distribution of E-cadherin upon CDKN2A expression. Interestingly, cells expressing p16INK4a tended to form large intracellular vacuoles upon suspension, and considerable amount of E-cadherin located on the vacuolar membrane (Fig. 3a). As the total E-cadherin showed little change (Fig. 3b), therefore CDKN2A/p16INK4a expression resulted in E-cadherin redistribution from cell surface onto vacuolar membrane, which conceivably weaken intercellular adhesion mediated by E-cadherin and CIC formation. Similarly, intracellular F-actin, as indicated by phalloidin staining (Fig. 3c), rearranged with increased cortical and less cytosolic localization (Fig. 3c, d) in p16INK4a-expressing cells (arrows indicated in Fig. 3c) as compared with control (enhanced green fluorescent protein (EGFP)-positive cells). Interestingly, increased cell volume likely diluted limited amount of F-actin (Fig. 4a) resulting in significantly reduced mean F-actin signal as indicated by line scanning quantification (Fig. 3d), which was believed to decrease cell stiffness and impart winner status to cells^15,16^ in competition assay.

### CDKN2A confers cells winner identity

To examine the effects of CDKN2A on identity determination, we first checked the levels of β-actin and p-MLC, two key components of actomyosin that are essential for CIC formation. Although little changes in total level were detected for β-actin, CDKN2A (p16INK4a and p14ARF) expression remarkably inhibited phosphorylation of MLC (Fig. 4a). Thus, CDKN2A imposed a negative role in contractile actomyosin, which is comprised of actin filaments and myosins. Previous work demonstrated that lower RhoA-regulated actomyosin contraction render cell more deformable to be winner during competitive CIC formation^15,17^, we therefore analyzed CIC structures formed between control and CDKN2A-expressing cells. As expected, CDKN2A (p16INK4a/p14ARF)-expressing cells are more likely to be outer cell as winner engulfing control cells (Fig. 4b–d). Together, the data above support a model that CDKN2A negatively regulates CIC formation by inhibiting MLC phosphorylation, rearranging cytoskeletal actin filaments and reducing cell surface E-cadherin.

### CIC formation negatively correlates with p16INK4a expression in breast cancers

**Fig. 5:**
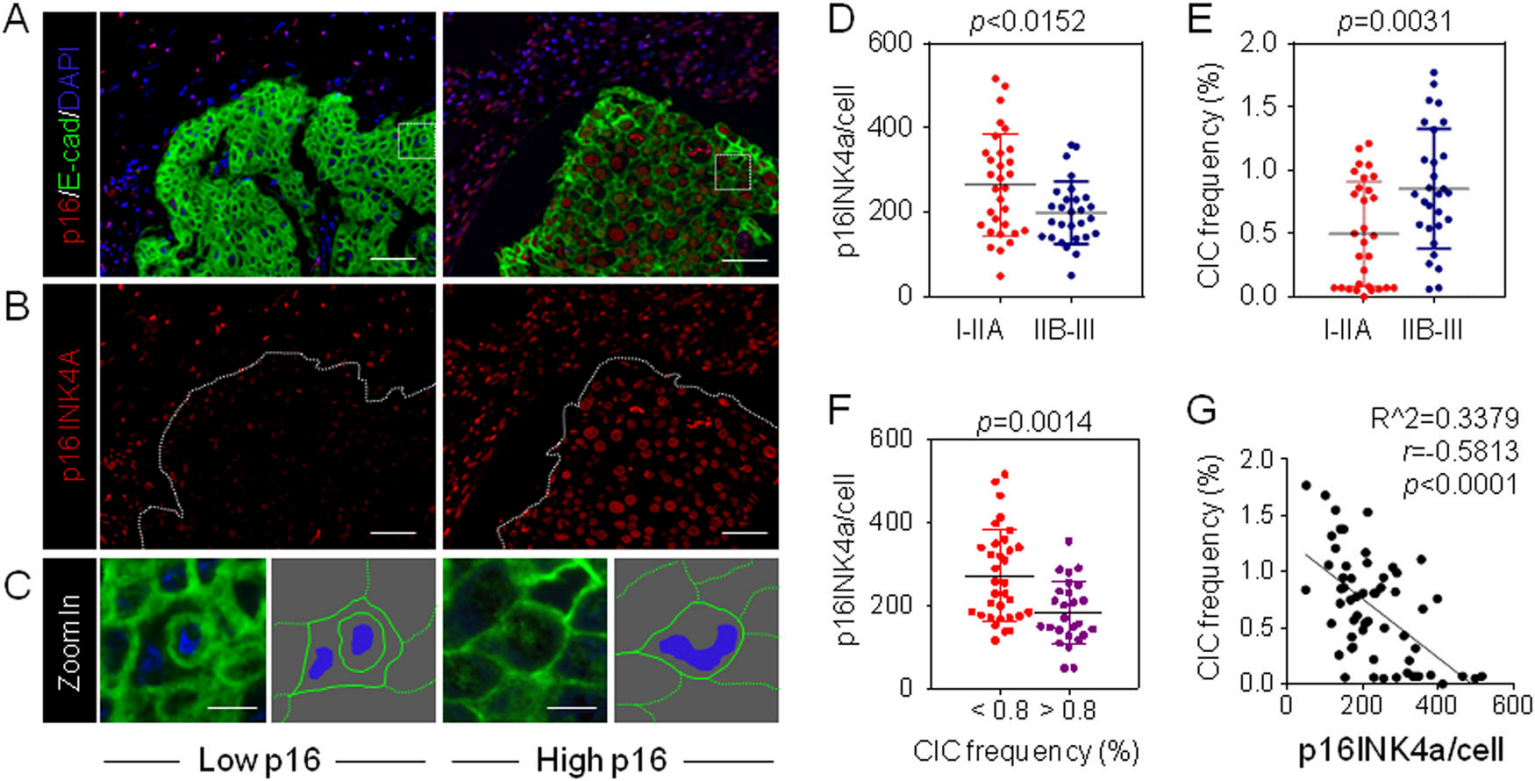
CIC formation negatively correlates with p16INK4a expression in breast cancers. **a** Representative images for p16INK4a and E-cadherin staining in human breast cancer tissues. Cancer cells are positive in E-cadherin. Scale bars: 50 μm. **b** Representative images for p16INK4a staining in **a**. The boundaries of Matrix cancer cells were indicated by dashed lines. Scale bars: 50 μm. **c** Zoomed images of boxed regions in **a**, right cartoons of each images depict morphologies of cells or CIC structures in the left. Scale bars: 5 μm. **d**, **e** Mean p16INK4a intensity per cancer cell (**d**) and CIC frequency (**e**) in staged human breast cancers. I-IIA: samples from stage I to IIA, *n* = 31; IIB-III: samples from stage IIB to III, *n* = 28. **f** Expression of p16INK4a in breast cancers of high CIC (>0.8%, *n* = 26) and low CIC (<0.8%, *n* = 33). **g** Correlation analysis between p16INK4a expression and CIC formation, *n* = 59

To further explore the clinical relevance of our finding, we examined p16INK4a expression and homotypic CIC formation, marked by E-cadherin staining, in a cohort of human breast cancer samples. As shown in Fig. 5a, b, p16INK4a and E-cadherin could be readily detected in patient samples. Although some cancer cells positive in E-cadherin express p16INK4a in levels comparable to those of adjacent matrix cells negative in E-cadherin (Fig. 5b, right), the others displayed significant decreased expression of p16INK4a (Fig. 5b, left), which tended to occur in cancers of higher TNM stage (Fig. 5d). Interestingly, CIC structures were more frequently detected in cancers of low p16INK4a (Fig. 5c, left and 5f) and high TNM stage (Fig. 5e). In agreement with a negative role of CDKN2A in CIC formation as proposed above, p16INK4a expression was inversely correlated with CIC frequency in human breast cancers (Fig. 5f, g).

## Discussion

Tumors are largely the results of uncontrolled cell proliferation and deregulated cell death. Interestingly, these two independent or even opposite cellular behaviors were found actually linked tightly^18^. On one hand, cell death, for example by apoptosis, could induce cell proliferation by caspase-initiated signaling cascades^19^; on the other hand, mitotic cell division frequently couples with cell death once went wrong^20^. As a non-canonical cell death process, CIC formation and subsequent cell death could sustain cell survival and proliferation under stressed condition such as starvation by providing outer cells with additional nutrients retrieved from dead inner cells^21^. Meanwhile, a portion of mitotic events could initiate entotic CIC formation in adherent cultures as demonstrated by Durgan et al.^22^ and our unpublished data. However, the molecular linker that directly couple mitosis and CIC-mediated cell death remains to be identified. In this work, we provided direct evidence demonstrating that CDKN2A is a potent inhibitor of CIC formation, therefore linking these two important cellular processes. As CIC formation will lead to the death of internalized cells, it is conceivable that activation of CIC-mediated cell death may function as a barrier for potential malignant transformation caused by inactivation of tumor suppressor genes such as CDKN2A. Consistent with the idea, inhibition of CIC formation could efficiently promote anchorage-independent growth of tumor cells^4,6,7^.

Given broad implications of CIC structures in multiple biological processes^2^, extensive efforts were endeavored to decipher the molecular regulation of CIC formation. In addition to the core elements of adheren junctions and actomyosin^5^, our recent work demonstrated that composition of membrane lipids had profound effects on CIC formation, high level of phosphatidylethanolamine, stearamide, lysophosphatidic acid and cholesterol negatively regulated CIC formation via inhibiting MLC phosphorylation^23^. And some liposomes, the routinely used transfection reagents, may influence the CIC formation in a cell-selective manner, which should be taken into account when interpreting data from liposome-treated cells^23^. Intriguingly, Hinojosa et al. found that Ezrin, the membrane-cytoskeleton linker regulated by MRTF-SRF signal, is essential for entotic CIC formation by sustaining membrane bleb dynamics^24^. Hamamn et al. found that glucose starvation could stimulate MCF7 cells to undergo entosis, which requires intact AMPK signaling to activate actomyosin contraction within the internalizing cells^25^. Together with our finding on cell cycle regulator CDKN2A, it seems that multiple signals that regulated cell survival and growth are intrinsically linked to CIC-mediated death, and cytoskeleton is likely the favorite target. It would be interesting to know whether, and how if there were, these multiple signals could crosstalk to each other under certain circumstances and contexts, which warrants detail analysis in the future.

## Materials and methods

### Cells and culture conditions

HEK293, ZR75-1, MCF7, 293FT cells, and their derivatives were maintained in Dulbecco’s modified Eagle’s medium supplemented with 10% fetal bovine serum (PAN-Biotech). MCF10A and its derivative cells were cultured as described^7^. For Dox-inducible gene expression experiments, inducible system transfected cells were cultured on the dish overnight and Dox was added to activate gene expression the next day.

### Antibodies and chemical reagents

Antibodies with working dilution factors, company source and catalog number include: anti-CDKN2A (1:1000 for western blot (WB); Abcam; ab3642), anti-MTAP-N (1:400 for WB; Santa Cruz; SC-17015), anti-β-tubulin (1:4000 for WB; CWBIO; CW0265), anti-Flag-tag (1:3000 for WB; CWBIO; CW0287), anti-HA-tag (1:3000 for WB; CWBIO; CW0092), anti-c-myc-tag (1:3000 for WB; CWBIO; CW0299), anti-p16INK4a (1:100 for immuno fluorescent staining (IF), 1:200 for IHC; BOSTER; BM1592/BM1924), anti-p14ARF (1:200 for IF; Santa Cruz; SC-S3640), anti-p-MLC (1:1000 for WB and 1:200 for IF; CST; #3671), anti-E-cadherin (1:1000 for WB and immunohistochemistry (IHC), 1:200 for IF; BD Biosciences; BD610182), anti-β-actin (1:5000 for WB; Sigma; A5441). Secondary antibodies include Alexa Fluor 568 anti-mouse (1:500; Invitrogen; A11031), Alexa Fluor 568 anti-rabbit (1:500; Invitrogen; A11036), Alexa Fluor 488 anti-mouse (1:500; Invitrogen; A11029) and Alexa Fluor 488 anti-rabbit (1:500; Invitrogen; A11034). Anti-rabbit IgG HRP (1:3000; CST; #7074), anti-mouse IgG HRP (1:3000; CST; #7076). Alexa Fluor®647 Phalloidin (1:200; Invitrogen; A22287) for F-actin labeling. 4,6-Diamidino-2-phenylindole (DAPI) was from Sigma (D8417).

### Constructs and stable cell lines

pQCXIP-EGFP-N1 was constructed as described^7^. The retroviral constructs for MTAP, p14ARF and p16INK4a were made in pQCXIP-EGFP-N1. pLVX-Lenti-X Tet-On Advanced vector was purchased from Addgene, p14ARF and p16INK4a were subcloned into pLVX-Tight-puro to generate pLVX-Tight-p16INK4a and pLVX-Tight-p14ARF. Stable cell lines were established by virus infection as described^7^. Cells were selected with appropriate antibiotics (1 µg/ml puromycin or 800 µg/ml G418 for MCF7, 2 µg/ml puromycin or 400 µg/ml G418 for MCF10A) for 7 days.

### Preparation of sgRNA and hCas9 expression vector

The bicistronic expression vector or precut pCS(puro) vector expressing sgRNA and hCas9 mRNA was gifts from Dr. Yongyi Xi (Institute of Biotechnology, Beijing). The BbsI-precut pCS was ligated with annealed oligos for CDKN2A, sgRNA-1: 5ʹ-caccGCACCGAATAGTTACGGTCGG-3ʹ and 5ʹ-aaacCCGACCGTAACTATTCGGTGC-3ʹ; sgRNA-2: 5ʹ-caccACCGTAACTATTCGGTGCGT-3ʹ and 5ʹ-aaacACGCACCGAATAGTTACGGTC-3ʹ; sgRNA-3: 5ʹ-caccGTGGGCCATCGCGATGTCGCA-3ʹ and 5ʹ-aaacTGCGACATCGCGATGGCCCAC-3ʹ. The presence of the inserted gRNA and stability of the final constructs was confirmed by sequencing. For transient transfection, HEK293 cells were transfected with CRISPR/Cas9 plasmids containing gRNA using Lipofectamine 2000 following the standard protocol and then cultured with regular medium for 48 h before analysis.

### CIC formation assay

Briefly, about 3 × 10^5^ cells were cultured in suspension in six-well plates coated with 0.5% soft agar for 6 h (HEK293 for 12 h). Cytospins were then made by centrifugation at 800 rpm for 4 min. And cells were then fixed and immunostained with phalloidin and DAPI to quantify CIC structures. Internalized cells wrapped at least half-way around by outer cells were considered as CICs.

### Competition assay

Stable MCF7 cell lines for pQCXIP-p14ARF or pQCXIP-p16INK4a were co-cultured in suspension with MCF7 cells transfected with control vector pQCXIP-EGFP-N1 for 6 h. Cytospin were fixed and stained as above in CICs formation assay. Cell’s identities were judged by EGFP positivity in CIC structures, those negative for EGFP were regarded as p16INK4a or p14ARF-expressing cells.

### Immunostaining and immunoblotting

For immunostaining, cytospins were first fixed in 4% paraformaldehyde and then preceded to routine staining with phalloidin and DAPI for 20 min before mounted with Prolong Gold antifade reagent (Invitrogen). Confocal images were captured and processed by *Ultraview Vox* confocal system (Perkin Elmer) on Nikon Ti-E microscope. For western blot, protein samples were subjected to sodium dodecyl sulfate–polyacrylamide gel electrophoresis and then transferred onto polyvinylidene fluoride membrane for standard immunoblotting.

### Tissue microarray (TMA) staining and image processing

A breast cancer TMA slide (HBre-Duc170Sur-01), purchased from SHANGHAI OUTDO BIOTECH CO. LTD, was stained with antibodies against p16INK4a and E-cadherin, and scanned by the Vectra® Polaris™ automated quantitative pathology imaging system (Perkin Elmer). Images were processed for tumor-matrix segmentation, intensity measurement, cell counting by inForm^®^ multispectral image processing software (Perkin Elmer) following standard instruction as described^1^. CIC was then quantified manually and defined as cellular structure with one or more cells fully enclosed within another cell of crescent nucleus. As CIC formation would lead to inner cell death, we therefore scored all structures displaying CICs morphology irrespective of the status (dead or live) of inner cells. Cell boundary was indicated by E-cadherin staining, which labels cell membrane. CIC frequency was calculated as CIC number divided by total cancer cells for each sample. TNM stage information was provided together with the TMA slide by SHANGHAI OUTDO BIOTECH CO. LTD.

### Statistics

All assays were carried out in triplicate or more. Data were expressed as means with standard deviations (SD). Pearson correlation was performed by correlation algorithm of GraphPad Prism software. *P*-values were calculated using two-tailed Student’s *t*-test from Excel or GraphPad Prism software, and *P*-values <0.05 were considered statistically significant.

## Electronic supplementary material

Patient information
